# Advances in Xmipp for Cryo–Electron Microscopy: From Xmipp to Scipion

**DOI:** 10.3390/molecules26206224

**Published:** 2021-10-15

**Authors:** David Strelak, Amaya Jiménez-Moreno, José L. Vilas, Erney Ramírez-Aportela, Ruben Sánchez-García, David Maluenda, Javier Vargas, David Herreros, Estrella Fernández-Giménez, Federico P. de Isidro-Gómez, Jan Horacek, David Myska, Martin Horacek, Pablo Conesa, Yunior C. Fonseca-Reyna, Jorge Jiménez, Marta Martínez, Mohamad Harastani, Slavica Jonić, Jiri Filipovic, Roberto Marabini, José M. Carazo, Carlos O. S. Sorzano

**Affiliations:** 1 Faculty of Informatics, Masaryk University, Botanická 68a, 60200 Brno, Czech Republic; 2 Spanish National Centre for Biotechnology, Spanish National Research Council, Calle Darwin, 3, 28049 Madrid, Spain; ajimoreno@gmail.com (A.J.-M.); jlvilasprieto@gmail.com (J.L.V.); eramirez@cnb.csic.es (E.R.-A.); dherreros@cnb.csic.es (D.H.); me.fernandez@cnb.csic.es (E.F.-G.); fp.deisidro@cnb.csic.es (F.P.d.I.-G.); pconesa@cnb.csic.es (P.C.); cfonseca@cnb.csic.es (Y.C.-F.R.); jjimenez@cnb.csic.es (J.J.); mmmtnez@cnb.csic.es (M.M.); roberto@cnb.csic.es (R.M.); carazo@cnb.csic.es (J.M.C.); coss@cnb.csic.es (C.O.S.S.); 3 Oxford Protein Informatics Group, Department of Statistics, University of Oxford, Oxford OX1 3LB, UK; ruben.sanchez-garcia@stats.ox.ac.uk; 4 Departament de Física Aplicada, Universitat de Barcelona (UB), Martí i Franquès 1, 08028 Barcelona, Spain; dmaluenda@ub.edu; 5 Departamento de Óptica, Universidad Complutense de Madrid, Plaza de Ciencias 1, 28040 Madrid, Spain; jvargas@ucm.es; 6 Institute of Computer Science, Masaryk University, Botanická 68a, 60200 Brno, Czech Republic; horacekj@mail.muni.cz (J.H.); davidmyska@mail.muni.cz (D.M.); 468922@mail.muni.cz (M.H.); fila@mail.muni.cz (J.F.); 7 IMPMC-UMR 7590 CNRS, Sorbonne Université, Muséum National d’Histoire Naturelle, 4 Place Jussieu, 75005 Paris, France; mohamad.harastani@upmc.fr (M.H.); Slavica.Jonic@upmc.fr (S.J.)

**Keywords:** Xmipp, Cryo-EM, Scipion, single-particle analysis

## Abstract

Xmipp is an open-source software package consisting of multiple programs for processing data originating from electron microscopy and electron tomography, designed and managed by the Biocomputing Unit of the Spanish National Center for Biotechnology, although with contributions from many other developers over the world. During its 25 years of existence, Xmipp underwent multiple changes and updates. While there were many publications related to new programs and functionality added to Xmipp, there is no single publication on the Xmipp as a package since 2013. In this article, we give an overview of the changes and new work since 2013, describe technologies and techniques used during the development, and take a peek at the future of the package.

## 1. Introduction

Xmipp is a software package for cryo-electron microscopy (Cryo-EM) and electron tomography (ET), available as a standalone project or via Scipion [1] framework. It offers multiple programs for almost all steps of the typical single particle analysis (SPA) processing pipeline and several programs for ET.

Originally, Scipion started from the graphical user interface of Xmipp, but it quickly branched off as a separate project of its own. At that time, Scipion and Xmipp [2] were available only as a single unit. Scipion was responsible for the inter-package operations between other programs and scripts, while Xmipp provided the programs, methods, and scripts for the actual processing.

Since 2018, Xmipp and Scipion are separate software packages. However, Xmipp is still providing some crucial functionality to Scipion and many auxiliary protocols that can be used in the processing pipeline of the Scipion project.

This article provides an overview of the work of the Biocomputing Unit of the CNB-CSIC, Madrid, concerning Xmipp. In the rest of the text, we use the term program to refer to the *Xmipp executable* and protocol to refer to the *Scipion protocol* provided by Xmipp. However, both expressions are interchangeable, as the executable is typically at the core of its respective protocol. Unless stated otherwise, this article refers to the latest Xmipp release available at the moment, i.e., version 3.21.06, released on 29 June 2021.

The article is divided into three parts. In Section 2, we overview the new programs and protocols added to the Xmipp package since its last dedicated publication in 2013. This section can also be interpreted as an overview of the most active research areas of the Biocomputing Unit of the CNB-CSIC, Madrid. For detailed information on each program or protocol, the reader is encouraged to visit the corresponding paper. This section also assumes that the reader has a general understanding of the SPA processing pipeline. Section 3 then talks about technologies and techniques used during the development. The last section summarizes our contribution to Cryo-EM, ET, and SPA in the last eight years and discusses possible future directions of the research.

## 2. New Programs and Protocols

**Figure 1:**
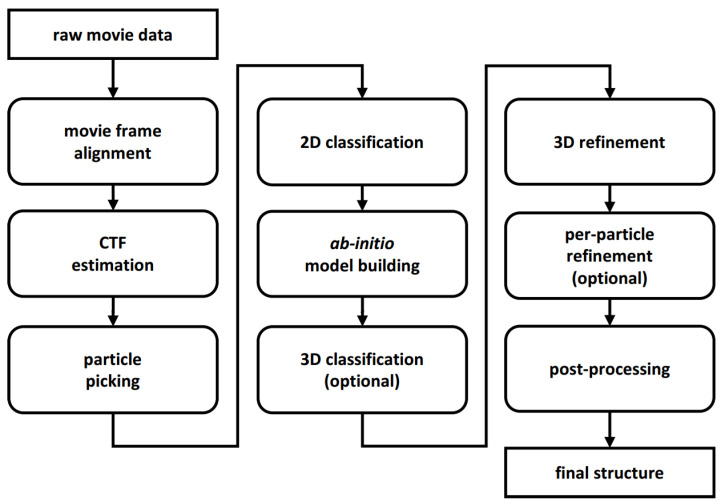
Typical steps of the Single Particle Analysis processing pipeline [3].

The image processing pipeline of the Cryo-EM project might be very complicated. However, it is typically divided into several general steps, as shown in Figure 1. In this section, we present new programs and protocols for Scipion added to Xmipp since 2013, thematically grouped.

### 2.1. Movie Acquisition and Frame Alignment

With the adoption of faster microscopes, the acquisition speed and the amount of the collected data steadily increases, and microscopes are expected to produce one super-resolution movie every few seconds soon. This creates high demands for (semi)automatic quality assurance and movie processing algorithms.

In 2016, in collaboration with the industry (Thermo Fisher Scientific), we proposed an image selection method using fast and efficient image quality descriptors computed during the acquisition that can be used to reject movies before further processing [4]. This algorithm was included in the current version of the EPU.

As for the frame alignment, in 2015, we designed a program for compensating the beam-induced motion called Optical Alignment, using Optical Flow (OF) [5]. The advantage of the OF is its parameter-free description of local movements, which gives it extremely high flexibility. In 2020, we presented FlexAlign [6], a GPU accelerated algorithm able to correctly compensate for the local shifts on the fly, using the current generation of hardware. This second algorithm sacrifices the flexibility of OF by using a small set of B-splines to describe the local movements. In practice, we have not found any significant difference between FlexAlign and Optical Alignment, meaning that the local deformation fields are sufficiently smooth. With this change, we have gained the possibility to store the local deformation with a minimal set of coefficients (as opposed to deformation fields twice as large as the movies themselves).

Xmipp also provides several other utility protocols, for example, the Movie Maxshift protocol for movie rejection based on the maximum shift of the corrected frames, the Split frames protocol for extracting only odd/even frames, the Movie Average protocol for creation of a simple movie average, or the automatic Movie Gain detection protocol [7] that can identify cases of incorrectly calibrated cameras. In addition, Xmipp provides the Preprocess Micrograph protocol for micrograph preprocessing, such as filtering or normalization.

### 2.2. CTF Estimation

There are multiple approaches to estimate the CTF of a given micrograph. For that reason, our group initiated the “CTF Estimation Challenge” [8] back in 2015 in collaboration with the National Center for Macromolecular Imaging (NCMI) at Houston. We have also designed the CTF consensus protocol, which can compare outputs of multiple CTF estimation algorithms. The CTF itself can be estimated via the CTF Estimation protocol, which we accelerated by using Zernike polynomials in [9].

### 2.3. Particle Picking

Particle picking is a challenging task, given the low Signal-to-Noise ratio of the input micrographs and the acquisition rate of modern microscopes. There are multiple approaches to detect particles. We used several new discriminative shape-related features and some statistical descriptions of the image grey intensities to train two support vector machine classifiers in the Particle Auto-Picking protocol for SPA [10].

In Random Conical Tilt and Orthogonal Tilt Reconstruction, particle picking is further complicated by the need to identify particle pairs, which we tried to address via Delaunay triangulation [11]. It can be found under Assign Tiltpairs protocol in Scipion.

Once the particle centers are known, particles can be extracted and further analyzed. In the Screen Particles protocol, we implemented a novel particle quality assessment and sorting method that can separate most erroneously picked particles from correct ones [12]. The Deep Consensus Picking protocol [13] utilizes a deep learning-based algorithm to lower the incorrectly picked particles by combining results of multiple pickers without any user intervention. We also used deep learning to detect carbon and other different types of high-contrast contamination in the Deep Micrograph Cleaner protocol [14].

In addition to the above-mentioned protocols, Xmipp provides several utility protocols, e.g., the Extract (Movie) Particles protocol for particle extraction from the micrograph or the movie, the Center Particles protocol for realignment of the uncentered particles, the Remove Duplicates protocol, the Screen Particles and Screen Deep Learning protocols for rejection based on several metrics or a deep learning model, or the protocol for Particle Boxsize estimation.

### 2.4. 2D Classification

2D classification is used to group similar particles into 2D classes, which are then filtered (to remove bad particles that the previous step has incorrectly identified as good ones) and used to generate the first 3D model of the sample at low resolution.

In 2014, we designed the CL2D protocol [15] for automatic 2D classification and outlier detection using a mixture between robust K-means and a hierarchical clustering algorithm. We showed that the core class (particles with low variation around the centroid of the homogeneous class) and the stable class core (a subset of class core images that is classified together in the classification hierarchy) could effectively remove contaminating particles. CL2D was accelerated via GPU in 2018 in the GL2D protocol. This GPU version of CL2D also includes the possibility of assigning particles to a certain class of a static set of classes on the fly.

In addition to CL2D, Xmipp provides protocols for 2D-alignment using a maximum-likelihood target function (ML2D) and the protocol for classification using Kohonen’s Self-Organizing Feature Maps (SOM) and Fuzzy c-means clustering technique (FCM) called KerdenSOM.

### 2.5. Ab-Initio Model Building

In 2014, we proposed a method based on an initial non-linear dimensionality reduction approach and random sample consensus [16], available via the RANSAC protocol. In 2015, we revised the fundamental mathematical expressions supporting Random Conical Tilt [17], that can be used to produce the initial structure. We also reformulated the initial volume problem within a weighted least squares framework, calculating the weights through a statistical approach based on the cumulative density function of different image similarity measures [18]. This work is available via the Reconstruct Significant protocol.

The most recent approach that we proposed [19] is a consensus protocol for the initial volume. It considers the whole population of initial volumes along with the experimental images. It allows the population to evolve according to the dynamics given by swarm optimization, thus avoiding user intervention. It can be used through the Swarm Consensus protocol.

To evaluate the quality of the 3D volumes, we suggested a statistical methodology that does not require tilt-pair images [20]. We further enhanced this method [21] to provide objective information about the precision and accuracy of each experimental particle image used in the reconstruction. These two methods are available through the Validate Nontilt and the Multireference Alignability protocols.

Xmipp also provides the Shift Particles protocol to correct the center of the particles in 2D if the 3D map compatible with them is shifted by any arbitrary amount in any direction.

### 2.6. 3D Alignment and Reconstruction

We introduced a gridding-based direct Fourier method for the three-dimensional reconstruction approach that uses a weighting technique to compute a uniform sampled Fourier transform [22] in 2015. In 2019, we accelerated this algorithm [23] as part of the extended collaboration with the High-Performance Computing research group at the CERIT-SC Centre in the Czech Republic. Both the CPU and GPU versions are available via the Reconstruct Fourier protocol.

While participating in the Map Challenge by the Electron Microscopy Data Bank, we developed the High-Resolution Reconstruction Protocol (HighRes) [24]. This protocol uses an approach similar to the standard projection matching with some important modifications, especially in detecting significant features in the reconstructed volume. HighRes was eventually accelerated using GPU in 2020.

We also helped with the evaluation of the Map Challenge [25] and we proposed a pair comparison method to sort reconstructions based on a figure of merit [26].

DeepAlign is our latest contribution towards 3D alignment [27]. We showed that the combination of deep learning and the classical projection matching approach could lead to improved reconstructions while decreasing the computational time.

In addition to the aforementioned protocols, Xmipp provides several utility protocols for volume (pre)processing, such as Preprocess volumes for thresholding or segmentation, the Filter Volumes for filtering, the Crop/resize volumes protocol, the Create|Apply 3D mask protocol, the Helical|Rotational Symmetry parameter estimation protocol, and the Validate overfitting protocol for checking how the resolution changes with the number of projections used for the 3D reconstruction.

### 2.7. 3D Classification

With the increasing resolution of the microscopes, automated data acquisition, and better and faster processing abilities, we can detect minor conformational changes in the examined sample. We participated in the web service 3DEM Loupe [28], which allowed for analysis of the reconstructed volume via Normal Mode Analysis (NMA). This service is no longer available. In 2014, we published a method on the detection of the continuous heterogeneity in Cryo-EM images and the visualization of these images in a conformational space of reduced dimension (Hybrid Electron Microscopy NMA, HEMNMA [29]), featuring easy-to-use and comprehensible graphical interface and the protocol in Xmipp [30]. This method is based on NMA of an atomic structure or a Gaussian-based representation of the reconstructed volume. The Gaussian-based representation of the reconstructed volume is described in detail and its performance fully evaluated in 2016 for NMA [31] and other tasks such as volume denoising in [32]. All work related to NMA is currently available via the ContinuousFlex plugin in Scipion [33], which is maintained by the group of Dr. Jonić.

The ContinuousFlex plugin currently contains the protocols required to run HEMNMA method (e.g., Convert to Pseudoatoms protocol, NMA Analysis protocol, NMA Alignment protocol, and NMA Dimred protocol) [33], StructMap method (Structure Mapping protocol) [34], and HEMNMA-3D method (Convert to Pseudoatoms protocol, NMA Analysis protocol, NMA Alignment Vol protocol, and NMA Vol Dimred protocol) [35]. The same Convert to Pseudoatoms and NMA Analysis protocols are called in both HEMNMA and HEMNMA-3D. The ContinuousFlex plugin additionally provides a protocol for synthesizing single particle images (Synthesize Images protocol) and a protocol for synthesizing subtomograms (Synthesize Subtomograms protocol) from a given atomic structure or an EM map. As these protocols can synthetize Cryo-EM and Cryo-ET data with several types of conformational distributions as well as without any conformational heterogeneity, they can be used for testing various methods, including those provided by ContinuousFlex plugin.

StructMap features a visualization technique that is based on a statistical analysis of distances among elastically aligned pairs of EM maps [34]. If one map is continuously deformed to fit the other map, we can visualize an arbitrary number of Cryo-EM maps as points in lower-dimensional distance space.

HEMNMA-3D is an extension of HEMNMA to analysing continuous heterogeneity in Cryo-ET subtomograms and includes missing-wedge compensation [35]. Each Cryo-ET subtomogram is analyzed in terms of conformational differences with respect to a reference (an atomic structure, a Cryo-EM map or a subtomogram average), independently from other subtomograms, which results in a conformational space of reduced dimension in which all subtomograms are visualized.

One of the main limitations after discrete 3D classification is that typically we obtain few majoritarian classes. These classes are capturing most of the particles and can be used to generate high-resolution maps. The rest of the 3D classes captured are usually minoritarian with low Signal-to-Noise ratios, which cannot be refined to high resolution. To increase the population of these minoritarian classes, we have recently proposed an approach to locally deform particles by the Optical Flow algorithm from one conformation to a different (but close) conformation, thus, increasing the number of particles of the minoritarian 3D classes [36]. This work is available via the Enrich protocol.

In 2016, we published a work on the automatic analysis of the forces associated with local deformations [37] available via the Calculate Strain protocol.

### 2.8. Sharpening, Denoising, and (Local) Resolution Estimation

Interpretation of the reconstructed volume can still be challenging due to the noise at high-frequency signal components. In 2016, we proposed denoising the EM maps using Gaussian functions [32]. This work is available via the Convert to Pseudoatoms protocol from the ContinuousFlex plugin for Scipion.

Local MonoRes protocol [38] is our method for local resolution estimation, which provides fully automatic and fast per-voxel resolution estimations. We later modified the algorithmic core of this MonoRes to deal with spatially variant noise and, therefore, estimate the local resolution in Electron Tomography. This algorithm is called MonoTomo [39] and, up to our knowledge, is the unique local resolution method for electron tomography.

In 2019, we proposed Local DeepRes, a deep learning 3D feature detection algorithm for local resolution estimation [40], and Localdeblur Sharpening [41], a fully automatic local sharpening method exploiting the local resolution information.

While local resolution provides a per-voxel estimation of the final resolution, it still does not provide information about resolution in specific directions. In 2020, we proposed MonoDir [42], which decomposes local resolution into the different projection directions, thus, providing a detailed level of analysis of the final map.

Our newest contribution is towards comparison of the Cryo-EM volumes. Current proposals to compare Cryo-EM volumes perform map subtraction based on adjustment of each volume grey level to the same scale. In [43], we present a more sophisticated way of adjusting the volumes before the comparison, which implies adjustment of grey level scale and spectrum energy, but keeping phases intact inside a mask and imposing the results to be strictly positive. The adjustment that we propose leaves the volumes in the same numeric frame, allowing to perform operations among the adjusted volumes in a more reliable way. This work is available in the development version of Xmipp and will be included in the next release via Volumes Adjust, Volumes Subtraction and Volume Consensus protocols.

### 2.9. Model Building

Partially related to model building is our contribution to the 3D model construction from the atomic structures using a very accurate conversion with Electron Atomic Scattering Factors [44]. It is available via the Convert PDB protocol.

In 2020, we contributed towards the inter-package integration of the model-building tools in Scipion [45] by adding several protocols, e.g., Extract Asymmetric Unit protocol or Export to DB protocol to help in the export process to the EMDB/PDB database. Note that to see these protocols, Scipion View has to be changed to the Model building.

To evaluate the quality of the map-to-model fit, we have proposed the FSC-Q measure [46] available via the Validate FSC-Q protocol, which is a quantitative estimation of how much of the model is supported by the signal content of the map.

### 2.10. Our Other Contributions and Xmipp Applications

We have used our knowledge of the SPA and many of the above-mentioned programs while processing data of multiple challenging structures. For example, we helped to reconstruct or analyze the VirE2-ssDNA complex [47], a bacterial multidrug homodimeric ABC transporter [48], human adenovirus light particles [49], polyhedral protein cages that efficiently self-assemble in vitro and in vivo [50], three-dimensional structure of paired C2S2M PSII-LHCII supercomplexes [51], oligomers of HsCPAP897-1338 [52], human RuvBL2 protein coding gene [53], human mAb–fHbp–mAb cooperative complexes [54], the flexibility and conformational dynamics of the infamous SARS-CoV-2 spike [55], and the triangular bipyramid fold comprising 18 coiled-coil-forming segments [56].

In 2014, we proposed a standard for transferring the information on the three-dimensional orientation between packages [57].

In 2017, we provided a detailed survey of the iterative reconstruction algorithms used in SPA and Electron Tomography [58]. In the same year, we also analyzed theoretical foundations and derivation of several concepts and thresholds used for resolution assessment in 3DEM [59].

In 2019, we published a survey of the analysis of continuous conformational variability of biological macromolecules [60] and reference analysis of the β-galactosidase using streaming in Scipion [61].

In 2020, we showed that global B-factor sharpening and deposition of only the sharpened maps in the Electron Microscopy DataBase could be detrimental [62]. In the same year, we also published a review of local resolution concepts and algorithms [63].

In 2021 we had a look at several issues related to data processing. In [64], we suggested that principal component analysis (PCA) is a useful tool to analyze flexibility, but only at low resolution. In [65], we analyzed the sensitivity to preferred orientations of several image processing algorithms used for angular assignment and 3D reconstruction. Then, we showed how to combine Xmipp and other plugins in Scipion to distinguish correctly from incorrectly estimated parameters of the processing pipeline to achieve a more confident assessment about the reconstructed structures [66]. Finally, in [67] we showed how Xmipp could be utilized with other protocols available via the Scipion framework in a complex processing pipeline. We also showed how combination of different packages and consensus tools can improve the resolution of the reconstructed volume. More specifically, the Plasmodium falciparum 80S Ribosome (EMPIAR entry: 10028, EMDB entry: 2660) with reported resolution of 3.2 Å has been reconstructed at 3 Å.

### 2.11. GPU Acceleration

Several of the Xmipp protocols and programs have their computationally intensive portions of the code accelerated via GPU using the CUDA Toolkit. The deep learning programs then use TensorFlow or Keras. The list includes the most performance critical protocols, such as CL2D (GL2D) [15], DeepAlign [27], RANSAC [16], FlexAlign [6], Projection Matching, Reconstruct Fourier [23], Reconstruct Significant [18], HighRes [24], Swarm Consensus [19], Split Volume, and Validate Overfitting protocol.

Programs using deep learning and the Optical Flow movie alignment can be executed both on CPU and GPU, though GPU is recommended for performance reasons.

We also use two additional tools to further optimize the performance of the GPU code. We have experimentally used the Kernel Tuning Toolkit (KTT) [68] to optimize the execution of several programs on the most commonly used GPUs. We also use the cuFFTAdvisor [69] to optimize the parameters used for the invocation of the cuFFT library.

### 2.12. New Programs and Protocols Summary

**Figure 2:**
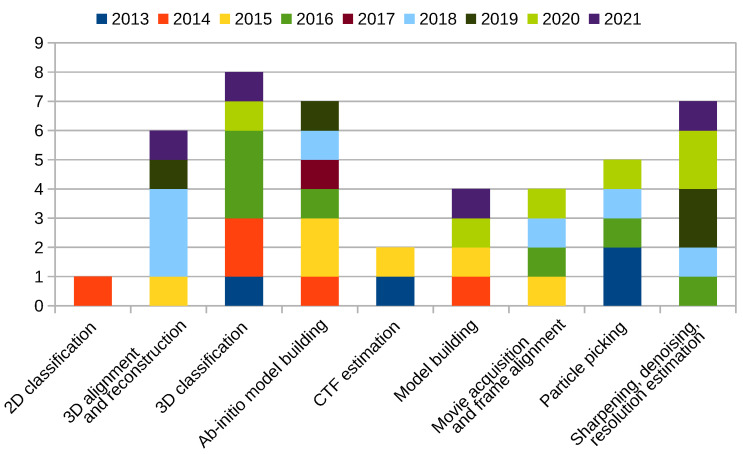
Xmipp related publications by category.

Figure 2 shows publications listed above, except those listed in the Section 2.10 [1,2,35]. As can be seen, the majority of contributions was towards the 3D classification and ab-initio model building, followed by 3D alignment and reconstruction and sharpening, denoising, and (local) resolution estimation. This is expected, as with the advances in the quality and amount of the input images, we need new techniques to fully utilize the information present in data. On the other end of the spectra, we have published only a single publication on the 2D classification implying that we no longer see 2D classification as a limiting factor.

**Figure 3:**
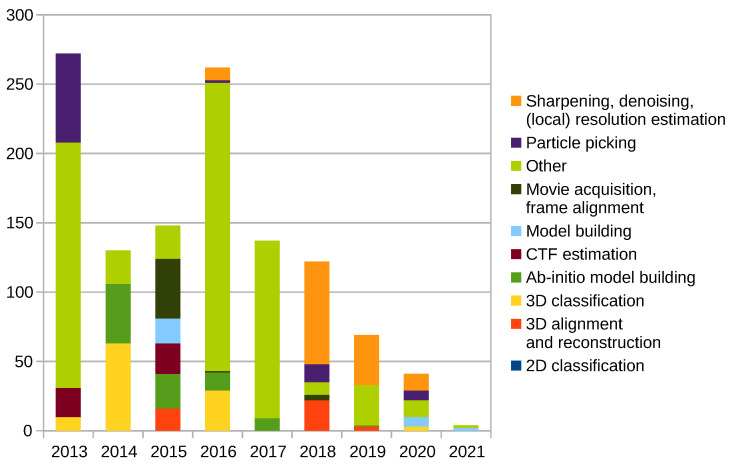
Citations of the Xmipp related publications by category.

One of the possible ways to measure the impact of the presented work is via citations. Figure 3 shows citations (As reported by Scopus, August 2021) of publications listed above, except those listed in the Section 2.10 and [35]. Our most cited papers, [2] and [1] with 208 and 165 citations, are also excluded. On average, we have over 14 citations per paper and over 63 citations on average per category. The most cited paper included in the figure is [38] with 74 citations, followed by [29] with 49 citations and [5,10,16] with 43 citations each.

**Figure 4:**
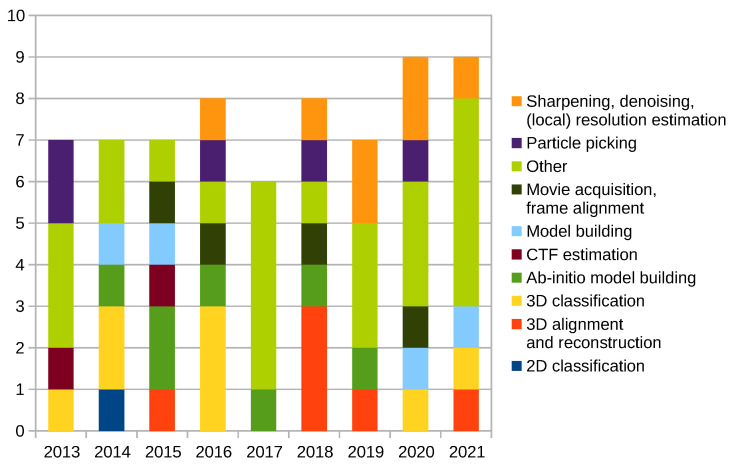
Xmipp related publications by year.

Figure 4 shows publications of the presented work by year, including those listed in the Section 2.10, [2] and [1], excluding [35]. On average, we publish or participate in over 7 papers per year.

**Figure 5:**
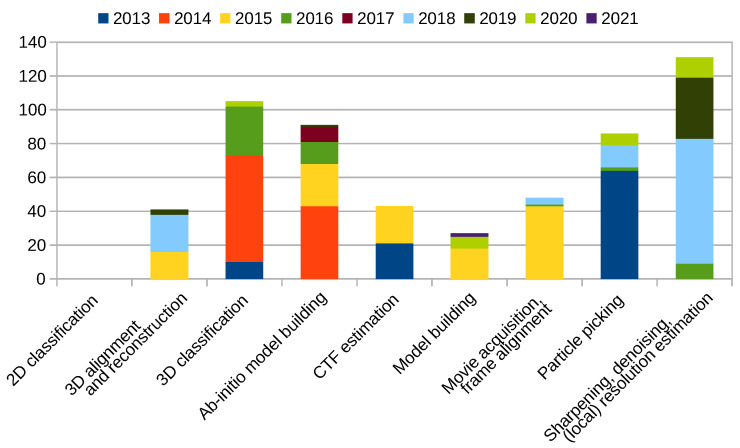
Citations of Xmipp related publications by year.

Figure 5 shows citations of Xmipp related publications mentioned above by the year of publishing, including those listed in the Section 2.10, [2] and [1], excluding [35]. As can be seen, both [2] and [1] had a huge impact on the Cryo-EM community.

#### Scipion Protocol Popularity

Scipion provides a list of the most used protocols at http://scipion.i2pc.es/report_protocols/protocolTable/. Provided that the user agreed with this data collection, each time the Scipion project is opened, a list of protocols used within this project is sent to our servers. This information is useful for checking which protocols are more used than others and concentrating on any performance issue related to those. This database currently holds information about over 25,000 workflows opened since November 2016.

At the time of writing this article (August 2021), Xmipp provided 37 out of the 100 most popular protocols. Out of them, the Manual|Auto Picking protocol [10], CL2D [15], HighRes [24], MonoRes [38], and several auxiliary protocols were the most used (each one has been used over 3000 times).

## 3. Technologies Used in Xmipp

As mentioned before, Xmipp is a suite of programs and (Scipion) scripts. It is a collaborative open source project hosted on GitHub, divided into four main repositories:

- Xmipp (https://github.com/I2PC/xmipp/) is the main repository.
- XmippCore (https://github.com/I2PC/xmippCore/) contains code responsible for data handling.
- XmippViz (https://github.com/I2PC/xmippViz/) contains code responsible for data visualization.
- Scipion-em-xmipp (https://github.com/I2PC/scipion-em-xmipp/) contains protocols for Scipion.

Historically, over 70 people participated in writing Xmipp. Currently, we version 786 C/C++ files (419,000 LOC (Lines of Code, comments excluded, including tests)), 278 Python files (55,500 LOC), and almost 200 Java files (31,100 LOC), contributing to the 290 executables and scripts used in 110 Scipion protocols.

Xmipp requires C++11 compatible compiler and JDK 11. Scipion protocols are written with Python 3.x. Xmipp provides Python binding, as well as optional Matlab binding. Optionally, Xmipp can use CUDA 8 to 11 and OpenCV versions 2 to 4. Xmipp uses SCons (https://scons.org/) as its construction tool.

We use multiple technologies to parallelize the execution of our binaries. In addition to MPI (https://www.open-mpi.org/) and built-in parallelization in Scipion, we use the CTPL library (https://github.com/vit-vit/CTPL) for multithreading, CUDA (https://developer.nvidia.com/cuda-toolkit) and cuFFTAdvisor (https://github.com/HiPerCoRe/cuFFTAdvisor) for GPU acceleration, and deep learning via TensorFlow (https://www.tensorflow.org/) and Keras (https://keras.io/). Experimentally, we also use StarPU (https://files.inria.fr/starpu/) for processing on heterogeneous machines and KTT (https://github.com/HiPerCoRe/KTT) for CUDA kernel optimization.

To ensure a certain quality of the code, we use a combination of unit testing via googletest (https://github.com/google/googletest), GitHub Actions for automatic project build, and static code analysis via SonarCloud (https://sonarcloud.io/organizations/i2pc/projects), pull request reviews, and integration testing via dedicated buildbot (https://buildbot.net/, http://scipion-test.cnb.csic.es:9980/)).

## 4. Summary

As can be seen, Xmipp has been heavily enhanced since its last publication in 2013. We have proposed, implemented, and provided to the community multiple algorithms for solving many steps of the SPA and ET processing pipeline.

There are three main general focus points of Xmipp.

1. High-quality results. As a general premise, we have favored accurate results over execution speed.
2. Automation of the data processing. The benefits include increased reproducibility and faster processing due to the minimization of manual intervention.
3. Consensus algorithms. By combining the results of multiple algorithms solving the same problem, we may verify the correctness of the answer.
4. Acceleration of the processing. Proper resource utilization and utilization of GPUs allow for much faster processing than just a few years ago.

We are also working hard to introduce new protocols for Electron Tomography, which is getting popular and a novel approach to conformational landscape analysis. Both will be accompanied by a publication once ready.

We also plan on improving the so-called meta-protocols, that is, protocols that create multiple intermediate protocols. These meta-protocols allow for fine-level control of the computation, such as the HighRes refinement or 3D classification of the input images.

In addition to the aforementioned papers, we are preparing a publication on approximating deformation fields to analyze continuous heterogeneity of biological macromolecules by 3D Zernike polynomials. This publication has been accepted and it is to be published soon.

We would also like to focus more on additional performance and resource utilization optimization as part of the long-term collaboration with the High-Performance Computing research group at the CERIT-SC Centre, Institute of Computer Science at Masaryk University in the Czech Republic.

## Sample Availability

Not available.
